# ALK alteration is a frequent event in aggressive breast cancers

**DOI:** 10.1186/s13058-015-0610-3

**Published:** 2015-09-17

**Authors:** Abdul K. Siraj, Shaham Beg, Zeenath Jehan, Sarita Prabhakaran, Maqbool Ahmed, Azhar R.Hussain, Fouad Al-Dayel, Asma Tulbah, Dahish Ajarim, Khawla S. Al-Kuraya

**Affiliations:** Department of Human Cancer Genomic Research, King Faisal Specialist Hospital and Research Center, Makkah Al Mukarramah Branch Road, Riyadh, 12713 Saudi Arabia; Department of Pathology, King Faisal Specialist Hospital and Research Center, Makkah Al Mukarramah Branch Road, Riyadh, 12713 Saudi Arabia; Oncology Center, King Faisal Specialist Hospital and Research Center, Makkah Al Mukarramah Branch Road, Riyadh, 12713 Saudi Arabia; Department of Pathology, Al-Faisal University, Al Zahrawi Street, Riyadh, 11533 Saudi Arabia

## Abstract

**Introduction:**

Breast cancer is the most common female malignancy worldwide and, despite improvements in treatment modalities, there are increased chances of recurrence and metastasis in a substantial number of cases and it remains one of the major causes of mortality among female cancer patients. Anaplastic lymphoma kinase (ALK) gene has been found to be altered in several solid and hematologic tumors. We aimed to comprehensively study the prevalence of ALK expression, and changes in copy number and translocation in a large cohort of breast cancer cases in a Middle Eastern population.

**Methods:**

ALK protein expression was investigated by immunohistochemistry and numerical and structural variations of the ALK gene were analyzed by fluorescence in situ hybridization (FISH) in a tissue microarray format in a cohort of more than 1000 Middle Eastern breast cancers. The data were correlated with clinicopathologic parameters and other important molecular biomarkers.

**Results:**

Immunohistochemical analysis showed ALK overexpression in 36.0 % of the breast cancer patients and gene amplification was present in 13.3 % of cases, seen by FISH analyses. ALK overexpression was significantly associated with ALK gene amplification (*p* = 0.0031). ALK-overexpressing tumors showed significant association with high-grade tumors (*p* = 0.0039), ductal histologic subtype (*p* = 0.0076), triple-negative phenotype (*p* = 0.0034), and high Ki-67 (*p* = 0.0001) and p-AKT (*p* <0.0001).

**Conclusions:**

Immunohistochemical analysis showed ALK is overexpressed in a substantial proportion of breast cancers and possibly plays a significant role in the aggressive behavior of this cancer. Gene amplification is hypothesized to be a possible cause for a significant proportion of this overexpression. Based on these findings, a potential role for an ALK inhibitor, as a therapeutic agent targeting aggressive subtypes of breast cancer, merits further investigation.

**Electronic supplementary material:**

The online version of this article (doi:10.1186/s13058-015-0610-3) contains supplementary material, which is available to authorized users.

## Introduction

Breast cancer is a heterogeneous group of diseases based on morphological features, molecular profiles, response to treatment and clinical outcome [1]. Every year, approximately 1.5 million women around the world are diagnosed with breast cancer [2]. It is the most common malignancy diagnosed among Saudi females [3] and is found to have an advanced stage, high grade and tends to affect a younger population as compared to the West [4, 5]. Despite improvement in treatment protocols and addition of new therapies, breast cancer continues to be the second leading cause of cancer mortality in women in the Western world [6]. Identification of new targeted therapy that allows progress in the management of breast cancer and improves survival is warranted.

Anaplastic lymphoma kinase (ALK), a tyrosine kinase receptor residing on chromosome 2p23 was first described in a subset of anaplastic large cell lymphoma (ALCL) patients as part of a chromosomal rearrangement with nucleophosmin as a fusion partner [7]. ALK has been reported to be translocated with other fusion partners, such as KIF5B [8], NPM1 [7], RET, ROS [9], VCL [10], TFG [11], EML4 [12] and MYH9, demonstrating its role in the pathogenesis of various cancers. The chimeric protein resulting from the fusion has lead to constitutively activated ALK tyrosine kinase [9, 10, 13]. Furthermore, other reports demonstrate additional modes of constitutively activated ALK kinase by mutations [14–16] and ALK gene amplification [17–19]. It has been suggested that the oncogenic role of ALK is most probably mediated via activation of tyrosine kinases that promote survival via activation of signaling pathways such as PI3-kinase/AKT [20] or by inhibition of apoptosis, thus leading to proliferation of cells. It has been demonstrated that inhibition of ALK inhibits growth of breast cancer cell lines and also tumor xenografts in mouse models [21].

ALK alterations such as increased ALK copy number, gene amplification and translocation have been shown to be present in 80 % of inflammatory breast cancer and 25 % of triple-negative breast cancers (TNBC), which are considered to be the most aggressive subtypes of breast cancers [21–23]. Moreover data generated from The Cancer Genome Atlas (TCGA) database on 479 breast cancer cases has also confirmed ALK deletions and copy number variations in breast tumors [21].

These known dysregulations in the ALK gene and their potential usefulness as biomarkers in many solid tumors, like inflammatory myofibroblastic tumors [24], esophageal squamous cell carcinoma [25] breast carcinoma [18] lung adenocarcinoma [9, 26] pediatric renal cell carcinoma [10] and neuroblastoma [27], have led to the development of ALK inhibitors and highlighted their therapeutic role in early clinical trials [18, 28, 29]. Crizotinib, an orally bioavailable tyrosine kinase inhibitor, has been shown to act against ALK kinase domain and is active against ALK-expressing tumors [24, 30, 31]. These recent studies have provided evidence for the emerging role of ALK as a potential molecular marker of diagnostic and therapeutic value in breast cancer.

Thus we attempted to investigate the structural and numerical alterations of ALK by fluorescence in situ hybridization (FISH) and protein expression by immunohistochemistry (IHC) in a large cohort of Middle Eastern breast cancers. We further correlated ALK alterations with clinical data including survival analysis, pathological parameters and other molecular markers in breast cancer patients.

## Methods

### Patient samples and data collection

One thousand and nine patients with breast cancer diagnosed between1990 and 2011 were selected from the files of the King Faisal Specialist Hospital and Research Centre (KFSHRC). The patients included in this study had their diagnosis, treatment and follow-up care in the Department of Surgical Oncology at KFSHRC. The histologic subtype of each breast tumor sample was determined according to World Health Organization (WHO) criteria. Waiver of consent was obtained for the study from the Institutional Review Board (IRB) and Research Ethics Committee (REC) of KFSHRC under Project RAC number 2040004 on breast cancer archival clinical samples. All samples were analyzed in a tissue microarray (TMA) format.

### Tissue microarray (TMA) construction

For each tumor, representative areas were selected mapped and a TMA was constructed using these mapped slides as a reference. The tissue microarrayer (Semiautomated Arrayer, CM1 Mirlacher, Neuenburg, Germany) was used and 0.6 mm diameter punches were obtained from the donor blocks. A map of the recipient block was prepared with coordinates and a number for each sample to correctly identify the tumor. The punched-out tissue cores from the donor block were inserted in the recipient block. The array blocks were incubated at 45 °C for 10 minutes to improve adhesion between cores and paraffin of the recipient block. They were cut at room temperature with a standard microtome (Thermo Shandon, Runcorn, UK) and slides were prepared using a tape-sectioning system (Instrumedics, Inc., St. Louis, MO, USA).

IHC and FISH staining were done on TMA slides cut from two replicas of TMA blocks for scoring to reduce the number of non-interpretable spots. Both the ALK IHC and ALK FISH non-interpretable spots were within the range of 10 % and the rest of the TMA spots were scored in concordance with earlier biomarker studies on TMAs [32].

### Fluorescence in situ hybridization (FISH)

FISH assay for ALK amplification and rearrangement was performed on a TMA format. The probe (BAC clone) corresponding to the ALK gene was selected by browsing Ensembl Genome Browser [33] and were purchased from the Children’s Hospital Oakland Research Institute (Oakland, CA USA). The BAC clone was cultured, DNA isolated and was labeled with digoxygenin (DIG) utilizing a DNA labeling kit from Roche (Roche, Hamburg, Germany). A commercially available centromeric probe for chromosome 2; CEP2 (Abbott Molecular, Abbott Park, IL, USA) was utilized as an internal control. FISH on a breast cancer TMA was performed as previously described [32]. Tissue samples were classified with an ALK/CEP2 ratio of 1.0 as normal; between 1.0 and 2.5 as having ALK gains; 2.5 and above as amplified [17]. A minimum of 25 cells were scored in a single microarray spot for the presence of both CEP2 and ALK signals. ALK gene amplifications and gains identified by the BAC clone RP11-328L16 were also further confirmed by the break-apart probe from Vysis (Abbott Molecular, Abbott Park, IL, USA) according to the manufacturer’s instructions using a BX51 Olympus fluorescence microscope (Olympus, Richardson, TX, USA). Briefly, in a normal cell with no gain or amplification, two fused red and green signals were observed. Increase in the number of red and green fused signals were considered to be amplifications and gains. An ALK break-apart probe set was also used to check for ALK gene rearrangement on breast cancer TMA slides. ALK rearrangement was termed as positive if >15 % of tumor cells showed split red and green signals and/or single red signals in addition to a single fused signal; otherwise, the specimen was classified as ALK FISH negative [26].

FISH analysis using the ALK probe was also performed on eight breast cancer cell lines (Cal-120, HCC-1937, EFM-19, HDQ-PI, CAL-51, MT-3, MDA-MB231 and MCF-10).

### DNA isolation

A Gentra DNA isolation kit (Gentra, Minneapolis, MN, USA) was utilized to extract DNA from paraffin-embedded breast cancer tissues using the manufacturer’s recommendations as described previously [34].

### Quantitative real-time PCR

The quantitative PCR (qPCR) technique was utilized to validate the copy number variation of the ALK gene observed by FISH. A few representatives DNA samples from formalin-fixed paraffin-embedded samples (FFPE) of breast cancer with normal and increased ALK copy number by FISH were selected for validation by quantitative real-time (qRT) PCR. DNA content was normalized to that of long interspersed element 1 (LINE1), a repetitive element for which copy number per haploid genome is similar in both the normal DNA sample and DNA from cancer cells. Primers were designed by Primer Express software v3.0 (Applied Biosystems, Foster City, CA) to hybridize to sequences of genomic DNA for ALK*.* The primers to the genomic sequences for ALK and LINE1 are as follows:

- ALK forward: CTT TGA CTT CCC CTG TGA GC
- ALK reverse: GCA GCC TCT CCC TTA CCT C
- LINE1 forward: CCG CTC AAC TAC ATG GAAACT G
- LINE1 reverse: GCG TCC CAG AGATTC TGG TAT G

The PCR conditions and Light Cycler PCR protocol were used as previously described [35]. The Pfaffl method for relative quantification was used to calculate the fold change for breast cancer samples that showed normal copy number and amplification of ALK gene [36].

### Immunohistochemistry

For IHC staining, TMA slides were processed and stained manually as described earlier [36]. A list of antibodies and their dilutions are listed in Table S1 in Additional file 1. For ALK IHC, primary ALK antibody (cloneD5F3; CST; 1:100 dilutions; Dako Target Retrieval pH9) was applied and incubated overnight. Visualization of the antigen antibody reaction was done using an enhanced polymer-based detection system, Envision Plus Dual Link System-HRP for 1 hour. Diaminobenzidene (Dako, Glostrup, Denmark) was employed for 5 minutes as the chromogen. A known CD30-positive ALCL case was utilized as a positive control and the negative control used was a mouse immunoglobulin G1 serum substitution for the primary antibody (ALK) [37]. The cutoff for estrogen receptor (ER) and progesterone receptor (PR) was taken as 1 % nuclear staining. Human epidermal growth factor receptor 2 (HER2) overexpression was assessed according to American Society of Clinical Oncology/College of American Pathologists (ASCO/CAP) guidelines [38]. The cutoff for high Ki67 was taken as more than 10 % nuclear staining [39]. For p-AKT immunoscoring only intensity scores were considered. Tumors with intensity 2+/3+ were considered positive and intensity 0/1+ were taken as negative [40].

For ALK IHC H-score (range 0–300) was obtained by adding the sum of scores obtained for each intensity and proportion of area stained [35]. X-tile plots were constructed for assessment of biomarker and optimization of cutoff points based on outcome as has been described earlier [41]. Breast cancers were categorized into two groups based on X-tile plots: one with complete absence (H score = 0) and the other with ALK expression (H score >0).

Two pathologists (SB and SP) independently performed IHC scoring for biomarkers and for discrepant scores consensus was established by reviewing the slides together.

### Cell lysis and immunoblotting

Proteins from MDA-MB231 and MCF-12A cells were isolated as described previously [42]. Twenty micrograms of isolated proteins were separated by SDS-PAGE and transferred to polyvinylidene difluoride (PVDF) membrane (Immobilon, EMD Millipore, Billerica, MA, USA). Immunoblotting was performed using ALK (Santa Cruz Biotechnology Inc., Dallas, TX, USA) and beta-actin (Cell Signaling Technology, Boston, MA, USA) antibodies and visualized by the enhanced chemiluminescence (Amersham, Pittsburg, PA, USA) method.

### Preparation of cytosolic and nuclear extract

MDA-MB231 and MCF12A cells were suspended in buffer A containing 10mM HEPES, 10mM KCl, 0.1mM EDTA, 0.1mM EGTA, 1mM DTT and the recommended amount of protease inhibitors and incubated on ice for 15 minutes. After incubation, cells were lysed with 10 % NP-40 and vortexed for 10 seconds. Cells were spun for 30 seconds at 14,000 rpm and supernatants containing nuclear-free cytosolic extracts were isolated. The remaining pellet was lysed in protein lysis buffer containing 0.4mM NaCl and nuclear extracts were isolated. Twenty micrograms of isolated proteins were separated by SDS-PAGE and transferred to polyvinylidene difluoride (PVDF) membrane (Immobilon, EMD Millipore, Billerica, MA, USA). Immunoblotting were performed using ALK and alpha-tubulin (Cell Signaling Technology, Boston, MA, USA) antibodies and visualized by the enhanced chemiluminescence (Amersham, Pittsburg, PA, USA) method.

### Statistical analysis

The JMP 10.0 (SAS Institute Inc., Cary, NC, USA) software package was used for data analyses. We examined the association of TNBC with clinicopathologic parameters, biomarker expression and also performed survival analysis. Survival curves were generated using the Kaplan-Meier method with significance evaluated using the Mantel-Cox log-rank test. Risk ratio was calculated using the Cox proportional hazard model. Values of *p* <0.05 were considered statistically significant.

## Results

### Clinicopathologic data

The clinicopathologic characteristics of the 1009 breast cancer patients are summarized in Table 1. The median age at the time of surgery was 46 years (interquartile range [IQR], 39.0–54.0 years). The median length of follow-up available for surviving patients was 53.0 months (IQR, 30–77 months). The 5-year overall survival for the study population was 80 %. The distribution of tumors by histologic type was as follows: 918 infiltrating ductal carcinomas (91.0 %), 46 infiltrating lobular carcinomas (4.6 %), 16 mucinous cancers (1.6 %) and 29 (2.9 %) others. TNM staging was performed as per WHO criteria. The majority of the patients were in stage II (37.7 %) at time of diagnosis followed by stage III (30.8 %), stage IV (9.1 %) and stage 1 (7.5 %).

**Table 1:** Clinicopathologic variables for the breast cancer patient cohort (n = 1009)

Age	
Median	46.0
Range (IQR)^	39.0–54.0
Histologic type	
Infiltrating ductal carcinoma	918 (91.0)
Infiltrating lobular carcinoma	46 (4.6)
Mucinous carcinoma	16 (1.6)
Other subtype carcinoma	29 (2.9)
Histologic grade	
Well differentiated	73 (7.2)
Moderately differentiated	484 (48.0)
Poorly differentiated	397 (39.3)
Unknown	55 (5.5)
Lymph node status	
Positive	627 (62.1)
Negative	306(30.3)
Unknown	76(7.5)
TNM stage	
I	76 (7.5)
II	378 (37.7)
III	314 (30.8)
IV	91 (9.1)
Unknown	150 (14.9)

^Interquartile range (IQR)

### ALK protein expression by IHC and correlation with clinicopathologic features

**Fig. 1:**
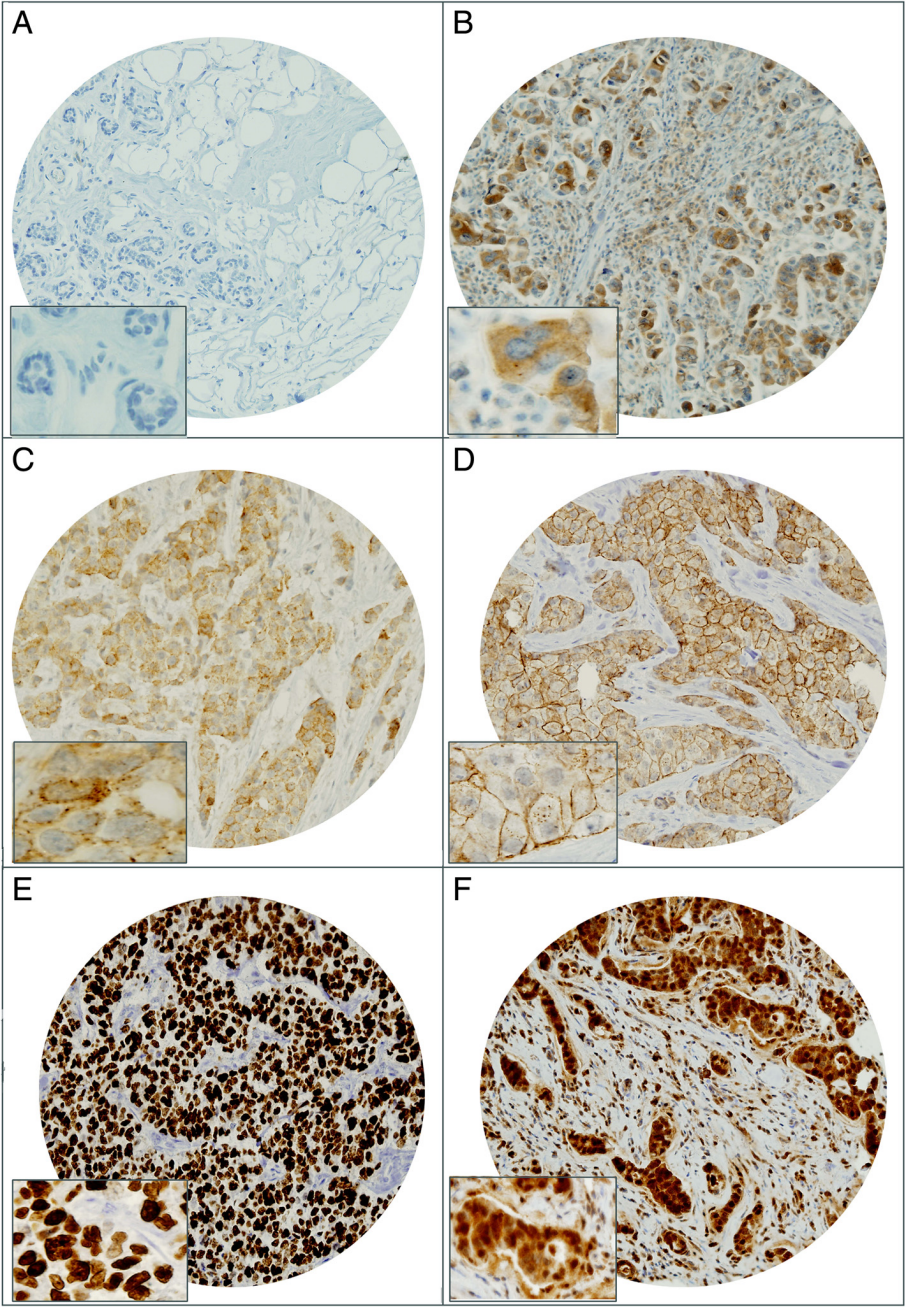
Tissue microarray-based immunohistochemical analysis of ALK in breast cancer patients. **a** Normal breast tissue showing negative ALK expression. **b** Breast cancer TMA spot showing ALK overexpression in cytoplasmic compartment. Inset is higher magnification (40×) view showing absence of nuclear staining. **c** Breast cancer TMA spot showing granular dot-like ALK staining in cytoplasmic compartment. **d** Breast cancer TMA spot showing membranous ALK staining in cancer tissue. **e** Breast cancer tissue array spots showing high proliferative index of Ki-67. **f** Breast cancer TMA spot showing p-AKT overexpression. 20 ×/0.70 objective on an Olympus BX 51 microscope (Olympus America Inc, Center Valley, PA, USA) with the inset showing a 40× 0.85 aperture magnified view of the same. *ALK* anaplastic lymphoma kinase, *TMA* tissue microarray

Of the 1009 breast cancer cases investigated, 36.0 % (350/972) of cases showed positive staining of ALK by IHC. Thirty-seven cases were non-interpretable due to loss of tissue cores or absent tumor cells in the core. The staining pattern ranged from an intense 3+ staining in the cytoplasm and/or membrane to 2+ moderate cytoplasmic staining and no cytoplasmic staining. A small subset of breast tumors (3.2 %) showed punctate coarse granular cytoplasmic staining (Fig. 1a–d). ALK expression was found to be significantly associated with poorly differentiated tumor (*p* = 0.0039), infiltrating ductal carcinoma subtype (*p* = 0.0076) and triple-negative breast cancer (*p* = 0.0034); however, no association was seen with age, lymph node involvement, distant metastasis and tumor stage. ALK expression was also found to be significantly associated with Ki-67 expression (*p* = 0.0001) and p-AKT (*p* <0.0001). (Table 2, Fig. 1e,f).

**Table 2:** Correlation of ALK IHC with clinicopathologic parameters in breast cancer

	Total		ALK present		ALK absent		*P* value
	N	%	N	%	N	%	
Total number of cases	972		350	36.0	622	64.0	
Age groups							
<50	663	68.2	250	37.7	413	62.3	0.1044
≥50	309	31.8	100	32.4	209	67.6	
Lymph nodes							
N0	300	32.9	108	36.0	192	64.0	0.5832
N1	294	32.3	105	35.7	189	64.3	
N2	192	21.1	69	35.9	123	64.1	
N3	125	13.7	53	42.4	72	57.6	
Metastasis							
M0	785	90.1	292	37.2	493	62.8	0.8336
M1	86	9.9	31	36.0	55	64.0	
Tumor stage							
I	75	8.9	20	26.7	55	73.3	0.0829
II	372	44.1	135	36.3	237	63.7	
III	311	36.8	130	41.8	181	58.2	
IV	86	10.2	31	36.0	55	64.0	
Histologic grade							
Well differentiated	74	7.7	21	28.4	53	71.6	0.0039
Moderately differentiated	493	51.2	159	32.2	334	67.8	
Poorly differentiated	395	41.1	156	42.0	229	58.0	
Histology							
Infiltrating ductal carcinoma	890	94.4	329	37.0	561	63.0	0.0076
Infiltrating lobular	40	4.2	7	17.5	33	82.5	
Mucinous	13	1.4	2	15.4	11	84.6	
Recurrence							
Yes	256	29.8	104	40.6	152	59.4	0.1225
No	602	70.2	211	35.1	391	64.9	
ER							
Negative	333	34.3	150	45.1	183	54.9	<0.0001
Positive	638	65.7	200	31.3	438	68.7	
PR							
Negative	412	42.5	178	43.2	234	56.8	0.0001
Positive	558	57.5	172	30.8	386	69.2	
Triple-negative							
Yes	147	15.2	69	46.9	78	53.1	0.0034
No	822	84.8	281	34.2	541	65.8	
HER2 FISH							
Amplified	275	29.0	108	39.3	167	60.7	0.2647
Normal/gain	672	71.0	238	35.4	434	64.6	
ALK FISH							
Amplified	126	13.3	61	48.4	65	51.6	0.0031
Normal/gain	821	86.7	284	34.6	537	65.4	
Ki-67 IHC							
High	818	85.8	318	38.9	500	61.1	0.0001
Low	135	14.2	30	22.2	105	77.8	
p-AKT							
Positive	212	22.6	132	62.3	80	37.7	<0.0001
Negative	725	77.4	211	29.1	514	70.9	
Molecular subtype							
HR+Her2-	460	47.4	142	30.9	318	69.1	0.0018
HR+Her2+	232	23.9	83	35.8	149	64.2	
TNBC	147	15.1	69	46.9	78	53.1	
HR-Her2+	132	13.6	56	42.4	76	57.6	
Survival							
OS 5 years				77.0		82.4	0.1212

*ALK* anaplastic lymphoma kinase, *IHC* immunohistochemistry, *ER* estrogen receptor, *PR* progesterone receptor, *HER2* human epidermal growth factor receptor 2, *FISH* fluorescence in situ hybridization, *TNBC* triple-negative breast cancer, *HR* hormone receptor, *OS* overall survival

*Data was not available (NA) for some cases: Age (NA = 19), Lymph nodes (NA = 74), Metastasis (NA = 101), Stage (NA = 145), Grade (NA = 50), Histology (NA = 29), ER (NA = 1), PR (NA = 2), Triple-negative (NA = 3), HER2 FISH (NA = 25), ALK FISH (NA = 24), Ki-67 (NA = 19), and p-AKT (NA = 35)

We also performed biochemical fractionation in breast cancer cell lines using nuclear and cytoplasmic extracts and found ALK expression to be more pronounced in cytosolic fraction (Figure S1 in Additional file 2) and had comparatively more expression in the triple-negative cell line compared to the non-tumorigenic epithelial breast cell line; MCF12A (Figure S2 in Additional file 3).

### ALK copy number alterations and translocations

**Fig. 2:**
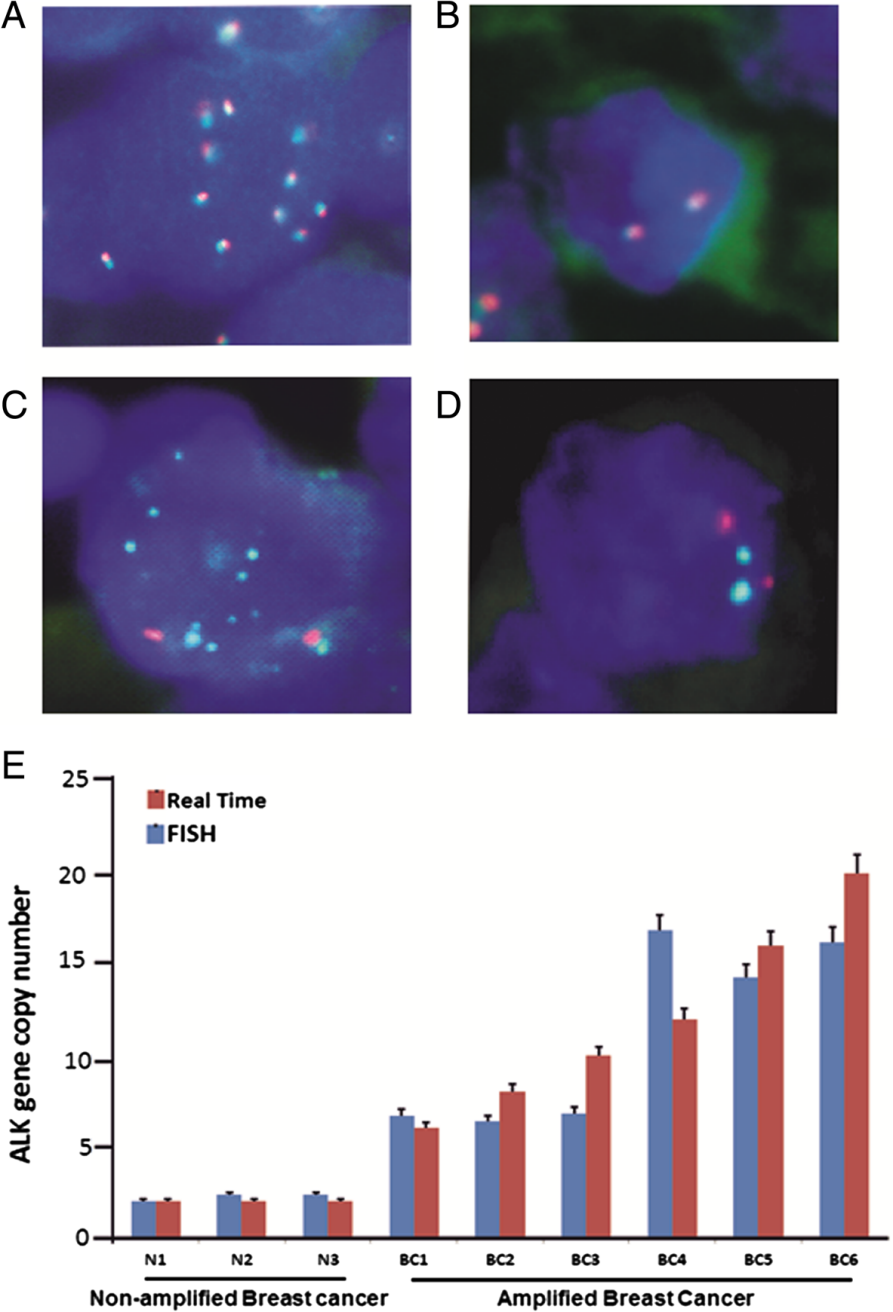
Determination of ALK gene copy number by fluorescence in situ hybridization. Copy number variation was further validated by an independent method of quantitative PCR in ALK amplified and non-amplified breast cancer samples. Tissues were screened on an Olympus BX 51 microscope (Olympus America Inc, Center Valley, PA, USA) under 100× magnification. **a** Breast cancer ALK amplified sample hybridized with ALK break-apart probe showing ten red and green fused signals. **b** Breast cancer ALK non-amplified sample hybridized with ALK break-apart probe showing normal tissue with two red and green fused signals. **c** Breast cancer ALK amplified sample hybridized with BAC probe RP11-328L16 and CEP2 probe showing two red CEP2 signals and seven to eight green signals representing the ALK gene amplification. **d** Breast cancer ALK non-amplified sample hybridized with BAC probe RP11-328L16 and CEP2 probe showing two red CEP2 signals and two green signals representing normal ALK copy number. **e** Verification of the ALK gene copy number by real-time quantitative PCR. Histogram showing ALK gene copy number obtained from normal breast samples 1–3 with two normal copies of ALK gene and samples 4–8 are amplified breast cancer samples. *ALK* anaplastic lymphoma kinase, *PCR* polymerase chain reaction

Numerical and structural abnormalities were analyzed by FISH in our cohort of 1009 breast cancer patients. A total of 980 spots out of 1009 showed interpretable results and 29 spots were non-interpretable due to absence of interpretable signals or loss of tissue spots. A total of 13.3 % cases (130/980) showed amplification of ALK gene (Fig. 2a–d). Amplification was observed in at least 30 % of the cells analyzed. We found ALK copy number gains in 70/980 (7.1 %) cases analyzed. In the majority of these cases, gains were seen in at least 30 % of the tumor cells. We also investigated the presence of EML4-ALK fusion by FISH and found no translocation of these two genes. In addition, we also did not observe any ALK gene amplification in six breast cancer cell lines (Cal-120, EFM-19, HDQ-PI, CAL-51, MT-3, and MCF-10) analyzed, however, two cell lines (HCC-1937 and MDA-MB231) showed amplification of the ALK gene.

The FISH data on breast cancer cases were further confirmed by qPCR analysis on few selected amplified and non-amplified breast cancer samples (Fig. 2e).

### Correlation of ALK gene copy number alteration and clinicopathologic features

Both ALK FISH and IHC data were available for 947/1009 cases. ALK gene amplification had a significant correlation with ALK protein expression (*p* = 0.0031) (Table 3). ALK gene amplification did not show any association with age, lymph node involvement, distant metastasis, molecular subtype of breast cancer and tumor stage. ALK gene amplification was significantly associated with mucinous histology (*p* = 0.0194) and Ki67 expression (*p* = 0.0129).

**Table 3:** Correlation of ALK FISH with clinicopathologic parameters in breast cancer

	Total		Amplified		Non-amplified		*P* value
	N	%	N	%	N	%	
Total number of cases	980		130	13.3	850	86.7	
Age groups							
<50	666	68.0	85	12.8	581	87.2	0.5018
≥50	314	32.0	45	14.3	269	85.7	
Lymph nodes							
N0	305	33.1	38	12.5	267	87.5	0.2010
N1	295	32.1	47	15.9	248	84.1	
N2	192	20.8	25	13.0	167	87.0	
N3	128	13.9	11	8.6	117	91.4	
Metastasis							
M0	789	89.7	111	14.1	678	85.9	0.5986
M1	91	10.3	11	12.1	80	87.9	
Tumor stage							
I	77	9.0	9	11.7	68	88.3	0.7588
II	374	43.8	57	15.2	317	84.8	
III	311	36.5	42	13.5	269	86.5	
IV	91	10.7	11	12.1	80	87.9	
Histologic grade							
Well differentiated	74	7.6	7	9.5	67	90.5	0.3393
Moderately differentiated	498	51.4	62	12.4	436	87.6	
Poorly differentiated	397	41.0	59	14.9	338	85.1	
Histology							
Infiltrating ductal	896	94.0	124	13.8	772	86.2	0.0194
Infiltrating lobular	44	4.6	1	2.3	43	97.7	
Mucinous	13	1.4	3	23.1	10	76.9	
Recurrence							
Yes	257	29.7	42	16.3	215	83.7	0.1569
No	308	70.3	77	12.7	531	87.3	
ER*							
Negative	332	33.9	48	14.5	284	85.5	0.4388
Positive	647	66.1	82	12.6	565	87.4	
PR*							
Negative	408	41.9	58	14.2	350	75.8	0.4010
Positive	566	58.1	70	12.4	496	87.6	
Triple-negative*							
Yes	141	14.5	16	11.3	125	88.7	0.4848
No	832	85.5	112	13.5	720	86.5	
HER2 FISH*							
Amplified	282	29.5	48	17.0	234	83.0	0.0418
Normal/gain	675	70.5	81	12.0	594	88.0	
ALK IHC*							
Present	345	36.4	61	17.7	284	82.3	0.0031
Absent	602	63.6	65	10.8	537	89.2	
Ki-67 IHC							
High	825	86.3	117	14.2	708	85.8	0.0569
Low	131	13.7	11	8.4	120	91.6	
p-AKT							
Positive	210	22.4	31	14.8	179	85.2	0.4232
Negative	729	77.6	92	12.6	637	87.4	
Molecular subtype							
HR+Her2-	461	47.2	58	12.6	403	87.4	0.6418
HR+Her2+	238	24.4	33	13.9	205	86.1	
TNBC	141	14.4	16	11.4	125	88.6	
HR-Her2+	139	13.9	22	16.2	114	83.8	
Survival							
OS 5 years				77.3		80.3	0.3303

*ALK* anaplastic lymphoma kinase, *FISH* fluorescence in situ hybridization, *ER* estrogen receptor, *PR* progesterone receptor, *HER2* human epidermal growth factor receptor 2, *IHC* immunohistochemistry, *HR* hormone receptor, *TNBC* triple-negative breast cancer, *OS* overall survival

*Data was not available (NA) for some cases: Age (NA = 27), Lymph nodes (NA = 72), Metastasis (NA = 100), Stage (NA = 144), Grade (NA = 53), Histology (NA = 27), ER (NA = 1), PR (NA = 6),Triple-negative (NA = 7), HER-2 FISH (NA = 23), ALK FISH (NA = 33), Ki-67 (NA = 24)

### Survival analysis

**Fig. 3:**
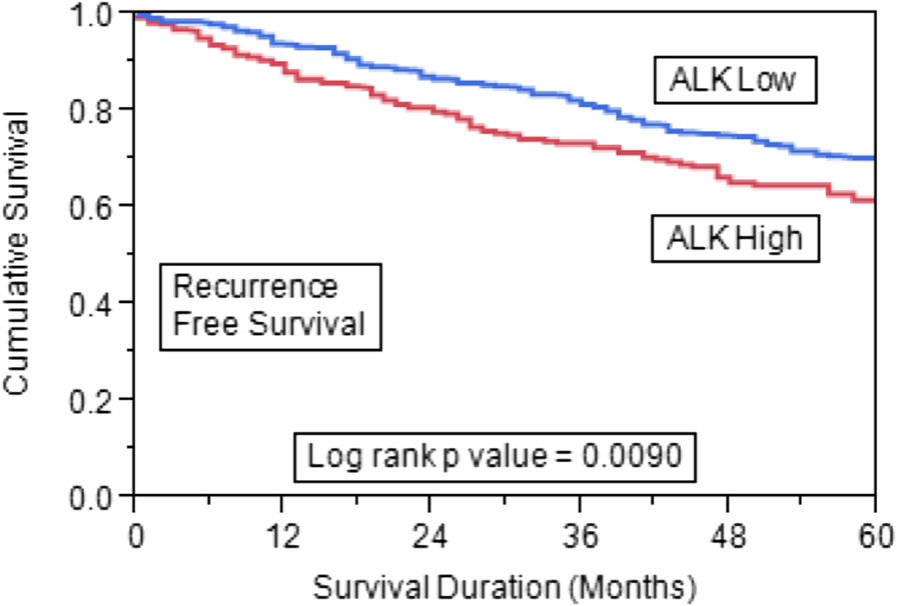
Kaplan-Meier survival analysis for the prognostic significance of ALK expression in breast cancer. Breast cancer patients with overexpression of ALK had reduced 5-year recurrence-free survival compared with those showing low expression of ALK (*p* = 0.0090). *ALK* anaplastic lymphoma kinase

The 5-year overall survival of our patient cohort showing ALK overexpression was lower than patients lacking this overexpression, however this survival difference could not reach significant statistical value (*p* = 0.1212, Figure S3 in Additional file 4). Recurrence-free survival was significantly lower for patients with ALK protein overexpression compared to tumors showing low ALK expression (*p* = 0.0090) (Fig. 3). ALK amplification was also not associated with any significant survival difference.

## Discussion

Breast cancer is one of the most extensively studied malignancies in the world and, despite improvement in the management of this cancer, it still remains a major cause of mortality and morbidity among female cancer patients [42]. Previously, reports have shown that ALK tyrosine kinase receptor is a strong biomarker and a good therapeutic target for a significant number of cancer patients [43, 44]. Therefore, in order to learn more on the prevalence and clinical significance of ALK overexpression and its association with clinical parameters in Middle Eastern breast cancer, we comprehensively investigated protein expression of ALK and numerical and structural alterations by FISH in a large cohort of breast cancer cases.

There is a wide variation in overexpression of ALK that has been reported in different subtypes of breast cancer. In this study, ALK overexpression was seen in 36 % of Middle Eastern breast cancer cases and this observation is in concordance with an earlier published study showing 47 % of ALK overexpression in a cohort of 100 breast cancer cases [45]. However, ALK overexpression as high as 75 % in infiltrating ductal carcinoma has been reported in a small cohort representing 63 samples from 22 patients [46]. Previously, Perez-Pinera et al. showed ALK overexpression in 50 % of the lobular subtype, whereby, we found ALK overexpression in only 17.5 % of lobular cases in our cohort [46]. Contrarily, Mehrjardi et al. could not find ALK overexpression in any case of lobular subtype in his patient cohort [45].

Significant association of ALK overexpression was seen with triple negative breast cancers (TNBCs). ALK protein was overexpressed in 47 % of TNBC cases, which was significantly higher than in non-TNBC cases. Our results show that the ALK signaling pathway possibly is more common in TNBC. Since TNBC is a poor prognosis breast cancer lacking any molecular target, so ALK overexpression can be exploited as a possible therapeutic target. ALK overexpression was also seen to be associated with poorly differentiated tumors (*p* = 0.0039) and tumors with a high proliferation index in our patient cohort, showing Ki-67 overexpression to be 42 % (*p* = <0.0001). The proliferation marker Ki-67 has repeatedly been confirmed as an independent predictive and prognostic factor in early breast cancer [47]. It has been shown that breast cancer with high Ki-67 expression responds better to chemotherapy [48], but is associated with poor prognosis [49]. This phenomenon is similar to the triple-negative paradox, which shows that TNBC had a poorer survival rate, despite a higher response rate to neoadjuvant chemotherapy [50]. In addition, TNBC has been shown to be associated with a higher expression of Ki-67 than non-TNBC [51]. These findings suggest that targeting ALK with its inhibitors might have promising therapeutic implications in treating such aggressive breast cancer subtypes especially TNBC, which has increased chances of recurrence and metastasis and poor clinical outcome due to absence of known hormonal and molecular targets.

ALK amplification was seen in 13.3 % of our breast cancer cohort. Previously, Tuma et al. had shown ALK amplification in 75 % of inflammatory breast cancer, however, the study was performed on a very small cohort of 12 cases [18]. The Cancer Genome Atlas (TCGA) dataset has also shown ALK amplification in 9 % of the breast cancer cases [52]. In addition we also sought for EML4-ALK translocation, which has been extensively reported in lung cancer [53]. Interestingly, we were not able to detect any translocation of ALK with the EML4 gene. Similar to our study, Fukuyoshi et al. also could not find this translocation in any of his 90 breast cancer cases tested for this translocation [54]. However, Lin et al. had shown the presence of EML4-ALK translocation in 2.4 % (5/209) of breast cancer cases [12]. Similarly, Robertson et al. had also found this translocation in one case out of 25 inflammatory breast cancer cases studied [21]. The exact cause of this discrepancy is not known, however, one possible reason could be the differences in ethnic population between studies as these authors had studied breast cancers from a Caucasian population while our study was conducted on Middle Eastern breast cancer cases.

ALK protein overexpression was significantly associated with ALK gene amplification in our study (*p* = 0.0031). Even though, there was significant association between ALK IHC and ALK FISH data but there was predominance in cases with protein overexpression compared to gene amplification (36 % versus 13.3 %). This difference could be partially attributed to the dependence of ALK expression on other signaling pathways, which had been previously hypothesized [55]. An alternative reason could be the presence of other translocation fusion partners with the ALK gene leading to ALK overexpression [56]. Moreover, the role of ALK mutations in causing protein overexpression cannot be ruled out as one of the possible mechanisms, as previously documented in neuroblastoma [57]. However, further studies are needed to explain these observations.

Another interesting observation was the strong association between ALK overexpression and p-AKT in our study (*p* <0.0001). The p-AKT signaling pathway is activated in different types of cancers [58]. This molecular correlation with the AKT pathway has also been shown previously in ALK-expressing ALCL [59]. The use of mTOR/AKT inhibitors along with ALK inhibitors have also been recently justified in ALK-altered neuroblastoma [60]. Therefore, it is hypothesized that ALK has a possible role in breast carcinogenesis via the AKT signaling pathway.

## Conclusions

In conclusion, we have comprehensively investigated the ALK alterations in a large cohort of breast cancer cases*.* ALK overexpression is present in a substantial proportion of breast cancer cases and is significantly associated with aggressive tumor parameters. ALK gene amplification accounts for a significant proportion of this protein overexpression and the role of other possible mechanisms are hypothesized. ALK inhibitors alone or in combination can be exploited as a novel therapeutic agent in aggressive subtypes of breast cancers. Further studies are required to validate these observations.

## Additional files

Additional file 1: Table S1. Details of primary antibodies used in the study. Details of primary antibodies used in the study including antibody clone, manufacturer and dilution used for each antibody. Additional file 2: Figure S1. Biochemical fractionation of ALK expression in breast cancer cell lines. (A) Nuclear-free cytosolic and nuclear extracts were isolated from MDA-MB231 and MCF12A cells and immunoblotted with antibodies against ALK and tubulin. (B) Relative expression of ALK was calculated using spot densitometry on cytosolic and nuclear immunoblots of MDA-MB231 and MCF12A cells. Expression of ALK was normalized with tubulin. Additional file 3: Figure S2. ALK expression in triple-negative breast cancer cell line versus normal cell line. (A) Total proteins isolated from MDA-MB231 and MCF12A cells were immunoblotted with antibodies against ALK and beta-actin. (B) Relative expression of ALK was calculated using spot densitometry on total protein immunoblots of MDA-MB231 and MCF12A cells. Expression of ALK was normalized with beta-actin. Additional file 4: Figure S3. Kaplan-Meier survival analysis for overall survival of ALK expression in breast cancer. Breast cancer patients with overexpression of ALK had reduced overall survival at 5 years compared with low expression of ALK, although not significant (*p* = 0.1212).

## Abbreviations

ALCL: anaplastic large cell lymphoma
ALK: anaplastic lymphoma kinase
ER: estrogen receptor
FISH: fluorescence in situ hybridization
HER2: human epidermal growth factor receptor 2
IHC: immunohistochemistry
LINE1: long interspersed element 1
PR: progesterone receptor
qRT-PCR: quantitative real-time polymerase chain reaction
TNBC: triple-negative breast cancer
