# Supplementary material for: ALK alteration is a frequent event in aggressive breast cancers
Source: Breast Cancer Res. 2015 Sep 17;17:127. doi: 10.1186/s13058-015-0610-3 (PMC4588266; doi:10.1186/s13058-015-0610-3)
Supplement: Additional file 1: Table S1. — Details of primary antibodies used in the study. Details of primary antibodies used in the study including antibody clone, manufacturer and dilution used for each antibody. [file 13058_2015_610_MOESM1_ESM.docx]

| **Antibody** | **Clone** | **Company** | **Dilution** | **Retrieval** | **Detection** |
| --- | --- | --- | --- | --- | --- |
| ER | 6F11 | NCL | 1:200 | pH6, pressure cooker | Envision+ |
| PR | 16 | NCL | 1:300 | pH6, pressure cooker | Envision+ |
| Her-2 | polyclonal | DAKO | 1:2000 | pH6, pressure cooker | Envision+ |
| ALK | *D5F3* | Cell Signaling | 1:100 | pH9, pressure cooker | Envision+ |
| Ki-67 | MIB-1 | DAKO | 1:500 | pH9, pressure cooker | Envision+ |
| p-AKT(Ser473) | D9E | Cell Signaling | 1:20 | pH9, pressure cooker | Envision+ |
|  |  |  |  |  |  |

**Supplementary Table 1: Details of primary antibodies used in the study.**
