# Supplementary figures and images for: ALK alteration is a frequent event in aggressive breast cancers
Source: Breast Cancer Res. 2015 Sep 17;17:127. doi: 10.1186/s13058-015-0610-3 (PMC4588266; doi:10.1186/s13058-015-0610-3)

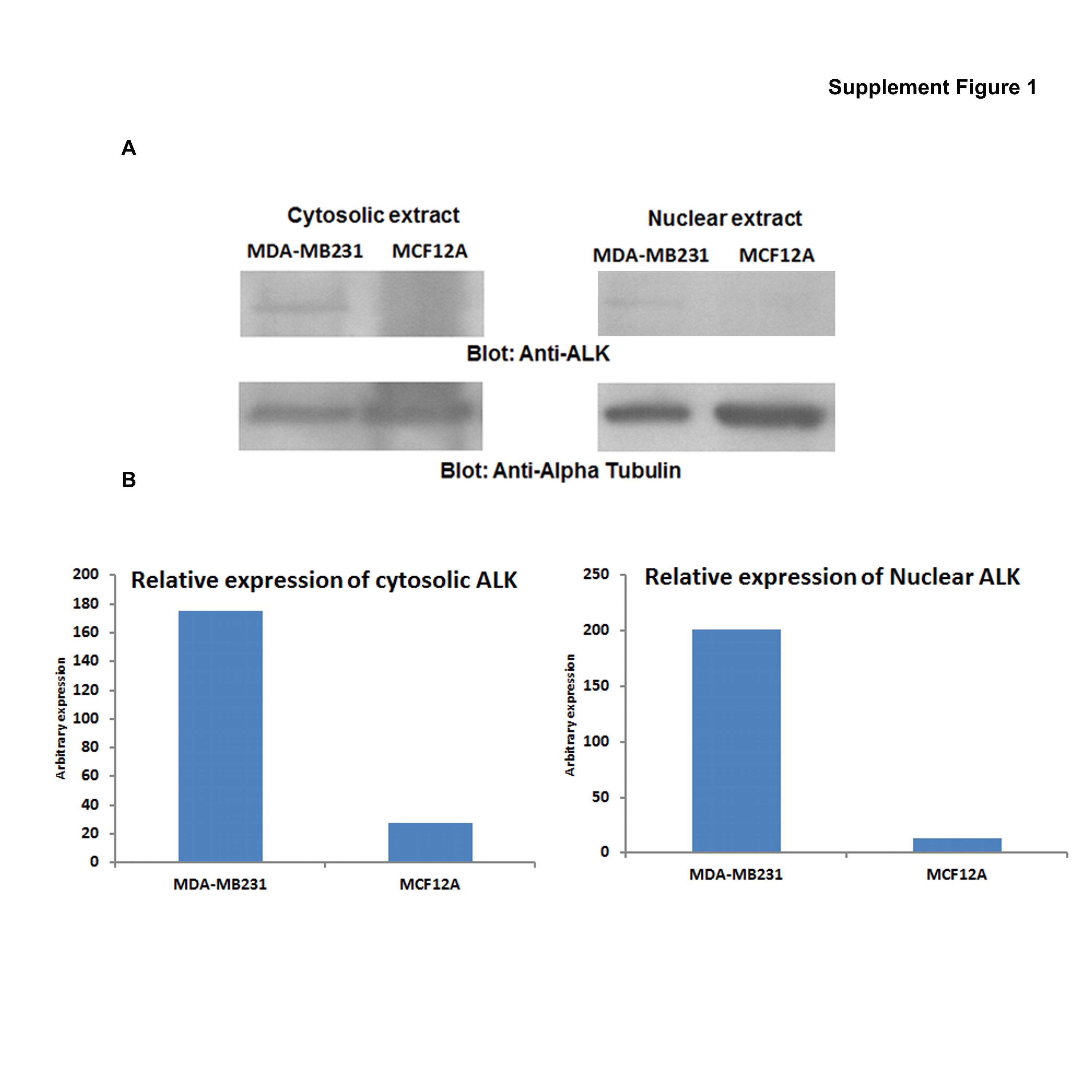

Supplement: Additional file 2: Figure S1. — Biochemical fractionation of ALK expression in breast cancer cell lines. (A) Nuclear-free cytosolic and nuclear extracts were isolated from MDA-MB231 and MCF12A cells and immunoblotted with antibodies against ALK and tubulin. (B) Relative expression of ALK was calculated using spot densitometry on cytosolic and nuclear immunoblots of MDA-MB231 and MCF12A cells. Expression of ALK was normalized with tubulin. [file 13058_2015_610_MOESM2_ESM.tif]

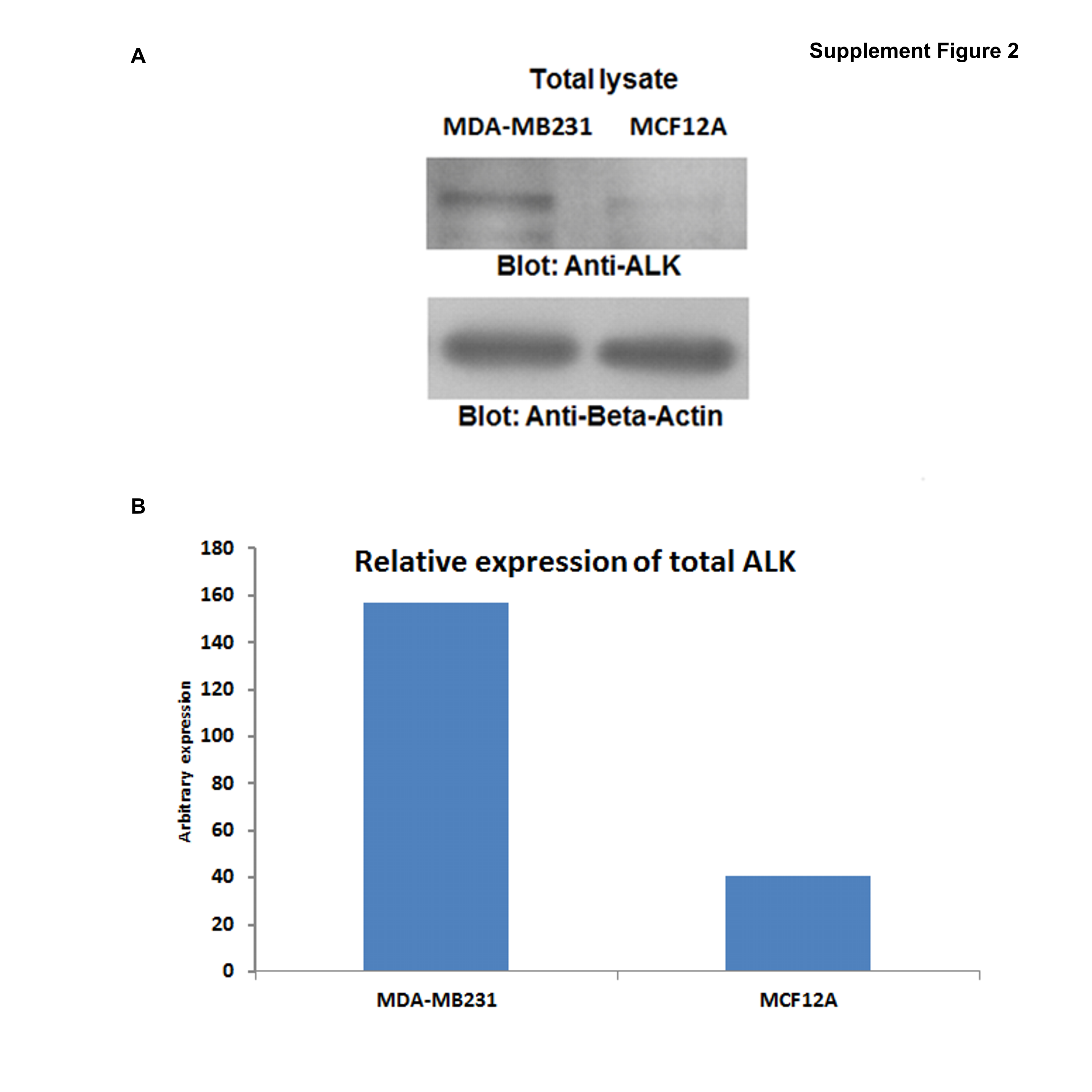

Supplement: Additional file 3: Figure S2. — ALK expression in triple-negative breast cancer cell line versus normal cell line. (A) Total proteins isolated from MDA-MB231 and MCF12A cells were immunoblotted with antibodies against ALK and beta-actin. (B) Relative expression of ALK was calculated using spot densitometry on total protein immunoblots of MDA-MB231 and MCF12A cells. Expression of ALK was normalized with beta-actin. [file 13058_2015_610_MOESM3_ESM.tif]

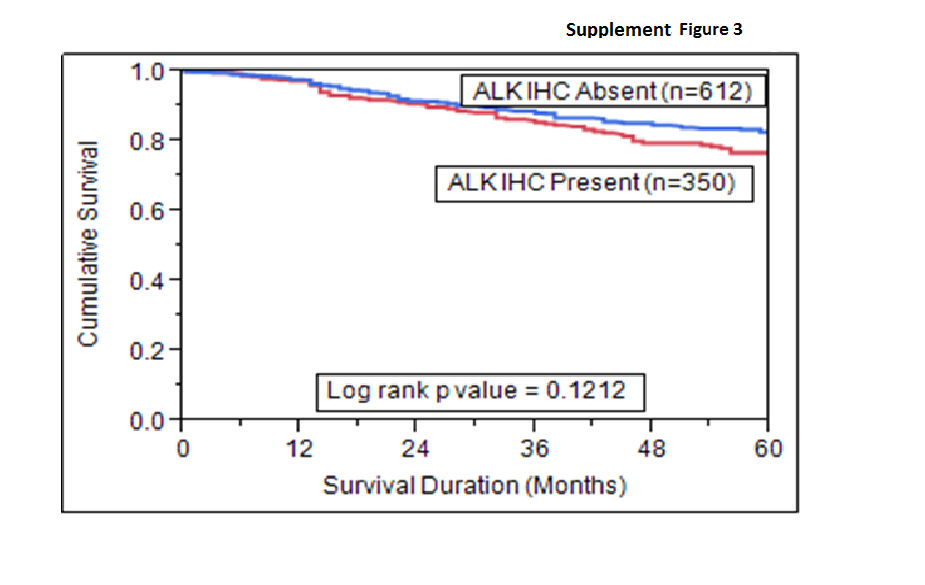

Supplement: Additional file 4: Figure S3. — Kaplan-Meier survival analysis for overall survival of ALK expression in breast cancer. Breast cancer patients with overexpression of ALK had reduced overall survival at 5 years compared with low expression of ALK, although not significant (p = 0.1212). [file 13058_2015_610_MOESM4_ESM.tif]
